# Do We Need Anti-Prionic Compounds to Treat Alzheimer’s Disease?

**DOI:** 10.3390/molecules24122237

**Published:** 2019-06-15

**Authors:** Dieter Willbold, Janine Kutzsche

**Affiliations:** 1 Institute of Complex Systems, Structural Biochemistry (ICS-6), Forschungszentrum Jülich, 52425 Jülich, Germany; j.kutzsche@fz-juelich.de; 2 Institut für Physikalische Biologie, Heinrich-Heine-Universität Düsseldorf, 40225 Düsseldorf, Germany

**Keywords:** Alzheimer’s disease, amyloid β, Aβ oligomers, anti-prionic, oral treatment, d-enantiomeric peptides

## Abstract

Background: While phase III clinical trials for the treatment of Alzheimer’s disease (AD) keep failing regardless of the target, more and more data suggest that the toxic protein assemblies of amyloid-beta protein (Aβ) and tubulin binding protein (TAU) behave like prions. Irrespective of the question of whether AD is theoretically or practically contagious, the presence of a self-replicating toxic etiologic agent in the brains of AD patients must have decisive consequences for drug development programs and clinical trial designs. Objectives: We intend to challenge the hypothesis that the underlying etiologic agent of AD is behaving prion-like. We want to discuss whether the outcome of clinical trials could have been predicted based on this hypothesis, and whether compounds that directly disassemble the toxic prion could be more beneficial for AD treatment. Method: We collected publicly accessible pre-clinical efficacy data of Aβ targeting compounds that failed or still are in phase III clinical trials. We describe the desired properties of an anti-prionic compound and compare it the properties of past and current phase III drug candidates. Results: We could not find convincing and reproducible pre-clinical efficacy data of past and current phase III drug candidates on cognition other than in preventive treatment settings. The desired properties of an anti-Aβ-prionic compound are fulfilled by the drug candidate RD2, which has been developed to directly disassemble toxic Aβ oligomers. Conclusion: RD2 is the first anti-prionic drug candidate. It is able to enhance cognition and impede neurodegeneration in three different transgenic AD mouse models, even under truly non-preventive conditions and even when applied orally. In addition, it is safe in humans.

## 1. Alzheimer’s Disease 

Alzheimer’s disease (AD) is a progressive neurodegenerative disorder which is associated with cognitive deficits, neurodegeneration as well as the aggregation of amyloid-beta protein (Aβ) and aggregation of TAU. The TAU protein (tubulin binding protein) is a microtubule-associated protein coded by the *MAPT* gene, which is localized in humans on chromosome 17 [1]. It exists in at least six isoforms originating from alternative mRNA splicing [2]. Mutations in the *MAPT* gene have been associated with several neurodegenerative diseases, but not with AD [3]. Aβ is a proteolytic fragment of the amyloid-beta precursor protein (APP) produced by beta and gamma secretase cleavages [4]. APP processing leads to various variants of Aβ, differing at their carboxy and amino-terminal ends (Aβ 1–39, Aβ 1–40, Aβ 1–42, and Aβ 1–43 as well as amino-terminal truncated and pyro-Glu-Aβ species) and with quite different aggregation propensities.

The resulting Aβ assemblies include soluble low molecular weight oligomers, protofibrils, and insoluble, fibrillar aggregates. Fibrillar Aβ is the major component of extracellular senile plaques, which are one of the histopathological hallmarks seen in the brains of AD patients.

There is overwhelming genetic data pointing to a decisive role of Aβ in whatever state, form or condition, in the development and progression of AD. Mutations in genes coding for the amyloid precursor protein APP or presenilin 1 (PS1), the catalytic part of the γ-secretase complex, often lead to early onset familial AD cases [5]. Mutations in the gene coding for TAU, however, do not lead to AD, but can lead to other disorders. Based on these findings more than 25 years ago, the amyloid hypothesis was formulated by Hardy and Higgins [6]. They postulated that accumulation of Aβ in the brain is the primary cause driving AD pathogenesis triggering all other neurodegenerative processes, including the formation of neurofibrillary tangles that lead to the loss of memory and other cognitive abilities [7]. On the basis of this theory, disease-modifying treatments were developed which should interrupt early pathologic events by reducing Aβ42 production (β- and γ-secretase inhibitors), increasing amyloid plaque clearance (immunotherapy) and decreasing plaque formation (Aβ_42_ lowering agents like tarenflurbil and inhibitors of amyloid aggregation tramiprosate), thus preventing later pathologic processes.

Despite this enormous scientific and economic effort, none of the drug candidates which were developed based on the amyloid hypothesis were successful in phase III clinical trials with beneficial effects on cognition decline. Currently, more than 20 million patients worldwide are affected which makes an effective therapy for AD perhaps one of the greatest unmet medical needs modern medicine is facing. At present the available medications (cholinesterase-inhibitors (ChEIs) and the N-methyl-d-aspartate (NMDA) receptor antagonist memantine) are only treating symptoms in a very limited way with unpleasant side effects [8] and are not able to slow disease progression. In addition, AD is not only a burden for patients, their relatives and care givers, but it also is a threat to healthcare systems. 

There is agreement that Aβ monomers are not toxic. Also, it is known for a long time already that plaque load does not correlate with AD progression [9]. Thus, especially over the past years, it has been postulated that the plaques themselves do not have the most deleterious effect, but rather small, mobile and soluble Aβ assemblies. Indeed, studies in recent years have strengthened the hypothesis that these so called Aβ oligomers are the major neurotoxic agent responsible for disease development and progression [10,11,12,13,14]. Furthermore, there is more and more evidence that Aβ oligomers, or at least sub-fractions of them, are able to replicate in a prion-like fashion [15,16,17]. 

Since Aβ-oligomers have been claimed to be the disease causing agent, there have been several attempts made to increase their clearance by passive or active immunization [11,18,19]. Although those attempts did show promising pre-clinical outcomes at least on plaque pathology and sometimes also on cognition under preventive treatment settings [20,21,22], none of these were beneficial for cognition in clinical trials yet [23,24,25]. Besides the missing efficacy to significantly decelerate cognitive decline or to ameliorate memory deficits, so-called amyloid-related imaging abnormalities (ARIA-E or ARIA-H) occurred in several cases (potentially indicating for example microhemorrhages or meningoencephalitis), representing some of the major severe side effects of Aβ-immunotherapy for Alzheimer’s disease [26,27], which is of concern especially because the prospective treatment is envisioned as life-long.

## 2. Aβ Aggregation

**Figure 1:**
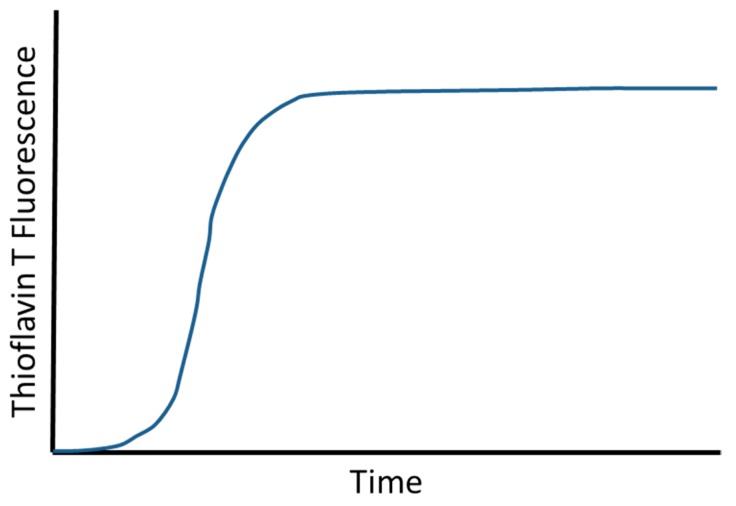
Scheme of a typical amyloid-beta protein (Aβ) aggregation assay result. Even at high micromolar Aβ concentrations, there is always a lag phase in ThT fluorescence detected Aβ aggregation experiments, followed by a steep increase in ThT-positive assembly formation and a saturation plateau at the end, when the monomer building blocks become depleted and limiting.

Aggregation of Aβ monomers into fibrils and other assembly species is thermodynamically favored. This is obvious, because otherwise it would simply not happen neither in the test tube nor in the brain. The formation of the first stable assembly is, however, kinetically very unfavored. This can be seen in every single Thioflavin T (ThT) fluorescence measured Aβ aggregation kinetic, which typically looks like the one shown in Figure 1. There is always a lag phase, followed by a steep increase in ThT-positive assembly formation and a saturation plateau at the end, when the monomer building blocks become depleted and limiting.

This kind of aggregation behavior has been described a thousand-fold and analyzed in great detail [28,29]. It very much resembles a bacterial growth curve. The steep (logarithmic) increase in ThT fluorescence indicates the presence of ThT-positive Aβ species that are capable for self-supported auto-catalytic growth and/or amplification of themselves. The big difference to a bacterial growth curve is of course that the start of the steep “growth” phase does not require inoculation with bacteria. This in turn suggests that the ThT-positive Aβ species capable of self-supported auto-catalytic growth and/or amplification of themselves can spontaneously form from Aβ monomers. This is supported by the observation that the length of the lag phase in the ThT assay, which starts with an initial period devoid of such Aβ assemblies can be drastically shorted by adding Aβ aggregate “seeds” that can be taken for example from other aggregation experiments at later stages of the experiment. But, as said in the beginning of this paragraph, this kind of Aβ aggregation behavior has been analyzed in much greater detail [28,29] and is described in a much more simplified version here only for educational and reasons and to prepare for the following thoughts on the implications for drug development and clinical trial designs. 

**Figure 2:**
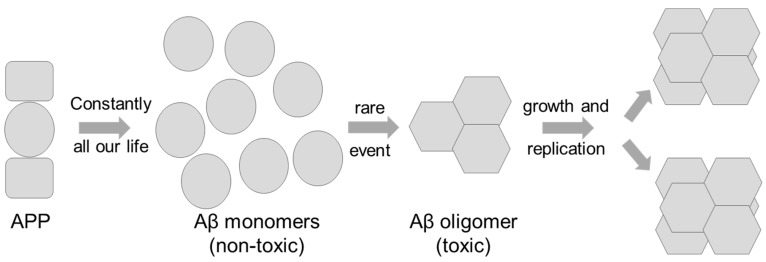
From left to right: Throughout our life time, Aβ monomers are constantly formed from the amyloid-beta precursor protein amyloid precursor protein (APP) by cleavage of beta- and gamma-secretases. Under physiological conditions (circa 1 nM Aβ) the formation of a stable Aβ oligomer is a very rare event, because several Aβ monomers have to meet in one place at the same time. This is a high order reaction, which is extremely dependent on the Aβ monomer concentration (e.g., to the sixth power). For rare things to happen, however, one only needs to wait long enough, explaining why life age is the most important risk factor for AD. Genetic disposition (familial AD cases and Trisomy 21) can lead to higher Aβ concentrations, making the formation of the first oligomer seed more likely to happen and thus occurs earlier in life. Once the first Aβ oligomer seed has been formed, it can further grow by consecutively consuming one Aβ monomer after the other. This is a kinetically favored first order reaction with a rate much less dependent on the Aβ monomer concentration. Aβ oligomers can break into smaller assemblies that grow again by consuming Aβ monomers. This is nothing else than a replication competent Aβ assembly containing Aβ monomer subunits in defined conformation, which is different from the intrinsically disordered native Aβ monomer conformation. In the scheme, different shapes symbolize different conformations of Aβ. For the sake of simplicity, the possibility of different Aβ assembly species containing Aβ monomers in even more different defined conformations has not been included in the scheme. This would be analogous to the concept of different existing prion strains with different properties. Please note that also the concept of secondary nucleation, which describes the oligomer-assisted seed formation in the vicinity of pre-existing assemblies, has not been included in this over-simplified scheme.

The critical concentration of Aβ for its aggregation has been described as 90 nM [30]. Thus, in order to investigate Aβ aggregation in vitro, it is necessary to study Aβ solutions in micromolar concentrations or even higher. Under physiological conditions (approximately 1 nM Aβ), aggregation is practically not observable, again underlining that the formation of the (first) Aβ seed is strongly kinetically unfavored. It also is line with the expectation that formation of the smallest stable Aβ assembly (seed) requires many monomer units to meet at the same time at the same place and thus is a high order reaction. Investigation of Aβ in a matrix-free solution yielded a pentamer or hexamer as the smallest populated and detectable assembly size [31], which nicely agrees with the “minimal fibril unit” of six Aβ monomer units defined in the high resolution Aβ fibril structure [32]. There have been reports on observed dimers, trimers, and tetramers as well, but those studies rely on sodium dodecylsulfate polyacrylamide gel electrophoresis (SDS PAGE) analysis of species with unknown SDS/Aβ ratios or on chemical cross-linking studies that would also allow other interpretations. For the following over-simplified scheme (Figure 2) and its description, however, the exact number of monomer units required for the formation of the minimal stable assembly is not even so important.

Accumulating evidence implicates a role for prion-like features in a number of neurodegenerative disorders, including AD. The etiologic agent responsible for the development and progression of the disease has been suggested to be a prion, prionoid or prion-like. There is more and more evidence that this assumption is principally correct [33,34]. This review does not intend to discuss or decide which name is the correct one. It is also not the right place to discuss whether this also means that the respective disease is contagious or not. The assumption that the Aβ aggregates underlying the disease progression behave like prions, however, has very important and decisive consequences for the development of a successful treatment strategy. For the rest of this review we will use the shortest possible name “prion” for such a replicating toxic Aβ assembly.

## 3. Did the Concept of a Replicating Toxic Aβ Assembly Predict the Outcomes of the Failed Phase III Clinical Trials?

Until now, we have witnessed many failures of compounds in clinical trials that were aiming to reduce Aβ formation (e.g., beta and gamma secretase inhibitors or modulators [35]) or compounds which increase the clearance of Aβ for example by passive or active immunization against different Aβ species [36]. Decreasing total Aβ levels may possibly be appropriate to make the formation of the first prion-like behaving oligomer less probable and thus to occur much later in life. Such a strategy, however, may only be preventively beneficial and thus “only” be potentially helpful to prevent or postpone the disease onset. 

Once Aβ oligomers have been formed and are already replicating in the brain, however, the reduction of total Aβ monomer substrates (by 20% to 30%), as envisioned by the use of beta- and gamma-secretase inhibitors and modulators, might in the best case slow down replication of prions, but is probably not enough to significantly slow disease progression. Taken from birth, beta- and gamma-secretase inhibitors and modulators could possibly delay the formation of the first Aβ prion seed. The situation in each AD patient’s brain is certainly post-seed-formation and prevention is too late. To the best of our knowledge, published pre-clinical data on past and current phase III beta- and gamma-secretase inhibitors and modulators have only shown efficacy on plaque load reduction. Beneficial effects on cognition have been obtained (if at all) only in purely preventive settings. Thus, based on the above described hypothesis that the etiologic agent for AD is a replicating toxic Aβ species, the failure of beta- and gamma-secretase inhibitors and modulators under non-preventive treatment settings—as it is in the patient—can, or even must be, expected to fail in the clinics.

The concept behind using anti-Aβ-antibodies (AAbs) is the expectation that they reach and find their target and induce its degradation by cells of the immune system (microglia and astrocytes) [37]. Obviously, Aβ monomer and Aβ fibril binding AAbs will not prevent Aβ oligomer prions from replicating. AAbs that are Aβ oligomer specific will, if they reach the target organ, bind to Aβ oligomers. They do not, however, destroy them. Thermodynamically, they will even stabilize them. AAbs rely on the immune system, which is not very efficient anymore in the target age group, anyway [38]. Much more importantly, it has not been shown so far that AAbs bound to Aβ oligomers have led to the ultimate disassembly or degradation of the bound oligomer. Further, AAbs are not very efficient in penetrating the blood brain barrier (BBB). Thus, it is not so surprising that to the best of our knowledge, published pre-clinical data on anti-Aβ-antibodies, which failed or are currently in phase III studies, have shown only efficacy on plaque load reduction. Beneficial reproducible effects on cognition have been obtained (if at all) only in preventive settings. This is exactly what one could have expected based on the above described hypothesis that the etiologic agent for AD is a replicating toxic Aβ species. Moreover, it is not clear what the disease relevant Aβ oligomer species looks like and whether a given antibody will bind it. Also, as it is known from prion protein strains, there might be an issue with the development of resistance to antibodies that are specific for a certain prion species [39]. 

Thus, with the current knowledge one could have already predicted from published pre-clinical data that treatment of AD patients with beta- and gamma-secretase inhibitors and modulators or with anti-Aβ-antibodies will probably not be beneficial for cognition, which is the crucial property for patient benefit and thus also for competent authorities for market admission and reimbursement. The most efficient ways to fight a replicating pathogen is either active immunization or applying substances that directly kill or destroy the pathogen, e.g., antibiotics in the case of bacterial pathogens. Active immunization against body’s own proteins, like Aβ has proven already, bears the risk of severe side effects.

Therefore, it is essential to directly eliminate Aβ oligomers by disassembling them into Aβ monomers or by converting them into other Aβ species that are not toxic.

It is obvious that the suggested therapeutic strategy to develop anti-prionic compounds and the underlying hypothesis of a self-replicating toxic protein assembly (prion) being the etiologic agent of AD needs to be evaluated in future proof of concept (PoC) studies in AD patients. The etiologic agent being a prion, however, would have predicted the clinical failures of the drug candidates tested so far in phase III.

Other approaches including anti-aggregation agents (β-sheet breakers e.g., HPYD or aggregation inhibitor like ALZ-801), anti-inflammatory agents (e.g., MLC901), Tau protein aggregation inhibitors (e.g., TRx0237), neuroprotective agents and autophagy inducers are currently in different phases of the Alzheimer’s disease drug development pipeline, for example reviewed in detail in Cummings et al. 2019 [40], on https://clinicaltrials.gov, https://www.alzforum.org or https://www.alz.org/. Their proof for a beneficial impact on the progression of the disease is still pending.

## 4. Consequences of the Concept of a Replicating Toxic Aβ Assembly as the Etiologic Agent of AD for the Potential Treatment of AD

What is essential for efficient disease treatment is the complete extinction of the toxic prion species. Thus, anti-prionics are needed that reach the brain and are able to directly destroy prion particles and disassemble them into natively folded monomeric Aβ molecules. 

Thus, we propose the development and application of anti-prionic compounds to treat prion diseases in general. What properties would such a compound need to have? 

**Figure 3:**
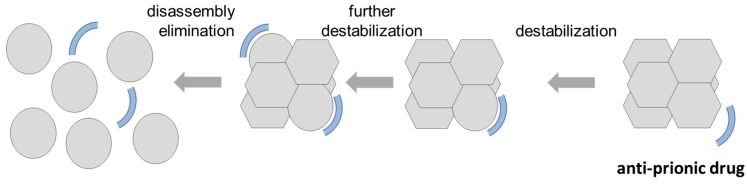
Suggested mode of action of an anti-prionic compound (from right to left): The desired anti-prionic compound should preferentially bind Aβ monomers and stabilize them in their native, intrinsically disordered conformation. This can be envisioned in a transient manner and is not necessarily in a 1:1 stoichiometry. When the compound is approaching the prion assembly, it will interact with one of the Aβ monomer subunits and thereby pushing its conformation towards the native one that does not perfectly fit to the rest of the prion particle. Thus, the prion particle is already destabilized a bit by this interaction. Each additional anti-prionic drug molecule will interact with further Aβ monomer subunits, further destabilizing and ultimately destroying and fully disassembling the Aβ prion particle. From a physicochemical and biophysical perspective, one would expect the action to be highly cooperative, clearly sub-stoichiometric (relative to the number of Aβ monomer subunits) and possibly even catalytic, because the drug molecules are not consumed during the prion destruction.

During the past years, we have therefore developed such an anti-prionic for Aβ prions. The compound RD2 was developed and optimized for stabilizing Aβ monomers in their native (intrinsically disordered) conformation to directly destabilize, disassemble and ultimately eliminate toxic Aβ oligomers via their direct disruption, rather than by relying on the immune system for their destruction. The mode of action is as follows (see Figure 3 from right to left). When the compound RD2 is approaching the prion assembly, it will interact with one of the Aβ monomer subunits, thereby trying to change its conformation towards the native one that does not perfectly fit to the rest of the prion. Thus, the prion particle is destabilized already. Each additional drug molecule will interact with more Aβ monomer subunits, further destabilizing and ultimately destroying and fully disassembling the Aβ prion particle. We have proven this kind of target engagement in vitro and shown that this oligomer disassembly is highly cooperative (Hill coefficient: 3) [41]. We have also shown that the drug candidate RD2 is efficient already at clearly sub-stoichiometric ratios. Both properties can be expected from this mechanism of action. 

The expected clinical impact/benefit of an anti-prionic therapy would be the deceleration or even stop of the cognitive decline and a partial recovery from cognitive dysfunctions. This would open up the possibility for patients to take care of their own daily living, as well as increase their length of live and especially their quality of life, which would be an enormous improvement for patients and their families. The socioeconomic impact will also be substantial, including cost reductions for healthcare systems. The idea to eradicate the replicative/infective agent during the treatment allows treatment periods that are not necessarily life-long. This would be of great advantage as life-long treatments have to be well-balanced between optimal efficacy and minimal undesirable side effects in order to maintain a high quality of life. Moreover, this approach aims to specifically eliminate all toxic oligomers regardless of any conformation or potential strain by stabilizing Aβ monomers in an aggregation incompetent conformation. 

We have confirmed this mechanism of action (MoA) additionally by a successful target engagement in vivo [42]. RD2 also has proven pharmacodynamical activity in several animal models carried out independently in multiple laboratories. Treatments with orally applied RD2 led to enhanced cognition in two different AD mouse models (APP_swe_/PS1ΔE9 and APP_SL_) [42,43]. Cognition improvement was even demonstrated in old-aged AD mice with severe cognition deficits and full-blown AD-associated pathology [42]. Furthermore, RD2 led to a significant deceleration of the motor neurodegenerative phenotype of pyroglutamate-Aβ expressing TBA2.1 AD-mice after oral administration [44]. 

In parallel, we were investigating potential alternative MoAs that could possibly also explain the beneficial pre-clinical treatment outcomes. One possible additional MoA would be the displacement of PrP from Aβ oligomers by RD2. It was shown previously that the cellular prion protein (PrP^C^) mediates toxicity of Aβ oligomers [45,46]. To examine if RD2 competes with PrP^C^ for binding to Aβ oligomers, we analyzed the Aβ(1–42)oligo/huPrP(23–230) hetero-assembly formation in the presence and absence of RD2 and revealed, that RD2 interferes with the hetero-assembly but only when Aβ(1–42)oligo is preincubated with RD2 before huPrP application [47], which has no relevance for a therapeutic treatment after disease onset.

A pharmacokinetic study using ^3^H-labeled RD2 yielded high oral bioavailability and penetration of the blood brain barrier [48]. In particular, oral administration resulted in a maximal brain concentration per dose (C_max_/D) of 0.06 (μg/g)/(mg/kg), a brain/plasma ratio of about 1, a bioavailability in the brain of 100% and very low ^3^H-RD2 levels in liver, kidney and plasma. Interestingly, in the brain ^3^H-RD2 was found after oral administration in amounts similar to all other tested administration routes (i.v., i.p., s.c.). Furthermore, RD2 showed a small elimination constant (0.0002 min^−1^) and long terminal half-life of 58 h in plasma. It also exhibits high bioavailability (F_AUC-28_) in plasma of about 76.5% [48]. In the liver and kidneys, oral administration resulted in a maximal concentration per dose (C_max_/D) of 0.09 (μg/g)/(mg/kg).

For D3, the lead compound of RD2, it was shown that adsorptive-mediated transcytosis (AMT) is involved in the transport of D3 across the BBB [49]. AMT has also been proposed for the transport of several arginine-rich peptides, such as the basic peptide derived from the human immunodeficiency virus type 1 (HIV-1) Tat proteins, across the BBB [50]. In extensive in vitro stability studies, we demonstrated that RD2 is remarkably resistant against metabolization in simulated gastrointestinal fluids, blood plasma and liver microsomes and shows no relevant human-specific metabolites [51]. Therefore, we concluded that it is perfectly suitable and safe for oral administration in humans. Safety was also investigated and proven in rodents and non-rodents in four-week toxicology studies (hemodynamic and respiratory parameters by telemetry), the Irwing test (motor, sensory, autonomic and integrative neurological function) and in a standard safety pharmacology panel including genotoxicity (AMES test, Micronucleus test), immunogenicity (ADA, T-cell mediated immunity) and a receptor screening. After finalization of all these relevant data, RD2 was recently successfully tested in a first-in-human first-in-class phase I clinical study (single ascending dose (SAD) Eudra CT # 2017-000396-93 and multiple ascending dose (MAD) Eudra CT 2018-002500-14). Both studies are completed and no drug-related adverse events (AEs) and no drug-related severe adverse events (SAEs) have been described.

In conclusion, the most straightforward consequences for drug development programs and clinical trial designs should be that the objective of the treatment must be the eradication or at least drastic diminishment of the toxic species. Once this is achieved, a life-long treatment is neither necessary nor attractive and this suggests trial designs that should reach their endpoints much faster than was envisioned in previous trials.

Therefore, the advantages of a strategy to treat AD patients with a compound that directly disassembles and destroys toxic replicating Aβ oligomer species by stabilizing Aβ monomers in their native intrinsically disordered conformation is a very promising treatment strategy and may well be the only efficient one. Such a treatment strategy does not at all intend to decrease the total Aβ levels in brain, plasma or CSF. It also does not intend to reduce plaque load, although this may be tolerated if achieved. With its mode of action, RD2 is the first anti-prionic drug candidate whose next important step will be a proof of concept in AD patients.
